# Plasticity of cerebellar Purkinje cells in behavioral training of body balance control

**DOI:** 10.3389/fnsys.2015.00113

**Published:** 2015-08-05

**Authors:** Ray X. Lee, Jian-Jia Huang, Chiming Huang, Meng-Li Tsai, Chen-Tung Yen

**Affiliations:** ^1^Department of Life Science, National Taiwan UniversityTaipei, Taiwan; ^2^Graduate Institute of Electronics Engineering, National Taiwan UniversityTaipei, Taiwan; ^3^School of Biological Sciences, University of Missouri-Kansas CityKansas City, MO, USA; ^4^Department of Biomechatronic Engineering, National Ilan UniversityIlan, Taiwan

**Keywords:** cerebellar cortex, neural plasticity, motor learning, reafference computation, information coding

## Abstract

Neural responses to sensory inputs caused by self-generated movements (reafference) and external passive stimulation (exafference) differ in various brain regions. The ability to differentiate such sensory information can lead to movement execution with better accuracy. However, how sensory responses are adjusted in regard to this distinguishability during motor learning is still poorly understood. The cerebellum has been hypothesized to analyze the functional significance of sensory information during motor learning, and is thought to be a key region of reafference computation in the vestibular system. In this study, we investigated Purkinje cell (PC) spike trains as cerebellar cortical output when rats learned to balance on a suspended dowel. Rats progressively reduced the amplitude of body swing and made fewer foot slips during a 5-min balancing task. Both PC simple (SSs; 17 of 26) and complex spikes (CSs; 7 of 12) were found to code initially on the angle of the heads with respect to a fixed reference. Using periods with comparable degrees of movement, we found that such SS coding of information in most PCs (10 of 17) decreased rapidly during balance learning. In response to unexpected perturbations and under anesthesia, SS coding capability of these PCs recovered. By plotting SS and CS firing frequencies over 15-s time windows in double-logarithmic plots, a negative correlation between SS and CS was found in awake, but not anesthetized, rats. PCs with prominent SS coding attenuation during motor learning showed weaker SS-CS correlation. Hence, we demonstrate that neural plasticity for filtering out sensory reafference from active motion occurs in the cerebellar cortex in rats during balance learning. SS-CS interaction may contribute to this rapid plasticity as a form of receptive field plasticity in the cerebellar cortex between two receptive maps of sensory inputs from the external world and of efference copies from the will center for volitional movements.

## Introduction

Physical activities involve both active and passive movements. Maintenance of balance is such a process, requiring integration of sensory information resulting from gravity-induced passive movement and self-generated active movement. Proper behavior requires animals to distinguish and respond differently to the same type of sensory inputs when they have different origins (i.e., active or passive movement) (Angelaki and Cullen, 2008; Cullen, 2011; Rochefort et al., 2013), such as reducing abnormal behavior like “ghost orienting” without a particular purpose or a specific stimulus (Anderson et al., 2012). This differential neural processing is thought to involve computational processing of sensory signals from peripheral receptors and motor command signals from motor centers to sensory receiving areas. The latter have been variously termed “efference copies” (Holst and Mittelstaedt, 1950), “corollary discharges” (Sperry, 1950), or “effort of will” (Helmholtz, 1866).

Neural sensory responses to active and passive activities are different in sensorimotor systems of a large number of species, such as the electrical sensory system of mormyrid fish (Zipser and Bennett, 1976; Bell, 1981; Requarth and Sawtell, 2014), the lateral line system of cartilaginous fish (Roberts and Russell, 1972), the escape response system of crayfish (Krasne and Bryan, 1973), the auditory sensory system in crickets (Poulet and Hedwig, 2003, 2006), the vocal-auditory system of songbirds (Prather et al., 2008), the vestibular system in monkeys (Roy and Cullen, 2001; Cullen and Minor, 2002; Brooks and Cullen, 2013), and the human somatosensory system (Blakemore et al., 1998; Mima et al., 1999). Effects of efference copies on sensory components from self-generated movement, termed “reafference” (Holst and Mittelstaedt, 1950), can be excitatory [e.g., dorsal giant interneurons of cockroaches (Delcomyn and Daley, 1979)], inhibitory [e.g., rostral fastigial neurons of monkeys (Brooks and Cullen, 2013)], or a combination of both [e.g., efferent neurons in the electrosensory lobe of mormyrids (Bell et al., 1997)]. In addition, results from both behavioral [e.g., perceptual learning of size-weight illusions in humans (Hershberger and Misceo, 1983)] and electrophysiological studies [e.g., spike-timing-dependent plasticity of mormyrid medium ganglion cells caused by central command signals (Sawtell et al., 2007)] imply learning dynamics involving computational processing between efference copies and reafference in the central nervous system. Juxtaposed with the neural plasticity underlying reafference and efference in learning is therefore the very role of plasticity processing sensory information during active and passive movements. Considering the neural plasticity during reafference learning, however, differential adjustment of neuronal responses to the same peripheral sensory inputs due to different causes (i.e., active or passive motion), here called “intention-dependent plasticity” (IDP), is still not well understood.

**Figure 1:**
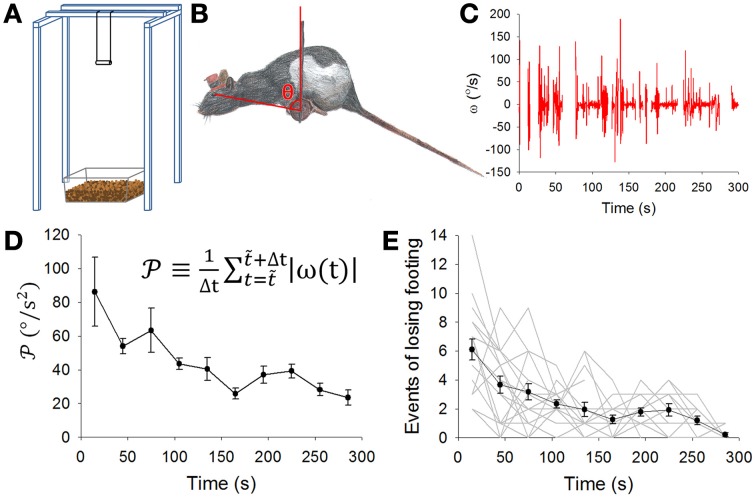
**Balance apparatus with the dowel and motor learning. (A)** The dowel was a wooden cylinder (8 cm long and 2 cm in diameter) hung 80 cm above the floor which was covered by a 5-cm layer of bedding material to cushion impact of the fall. **(B)** A rat balancing on the dowel. The pitch angle of head-body of the rat in the sagittal plane was defined as the angle θ between a line perpendicular to the ground and second line extending from the center of the dowel to the eye of the rat. **(C)** A typical record of the pitch angular velocity (ω, derived from the first time derivative of θ) while a rat was balancing on the dowel. **(D)** Pooled data (mean ± SEM, *n* = 18 rats) showing motor learning expressed as a gradual decrease in the level of movement 𝒫 [defined by the equation shown in **(D)**]. **(E)** Pooled data showing motor learning expressed as a gradual decrease in the number of times a rat lost its footing on the dowel. Gray lines are data from individual animals. Results in **(D,E)** indicate rat's motor learning on the balancing task.

Learning to maintain balance has long been hypothesized to involve neural plasticity in the cerebellum. According to Marr (1969), the cerebellar neuronal circuitry provides a cellular foundation for sensorimotor integration, with Purkinje cells (PCs) playing a central role in motor learning and/or acquisition of conditioned responses by interactively processing central instructive signals and peripheral sensory inputs. Based on neuronal responses recorded from different areas of the vestibular system in monkeys (Roy and Cullen, 2001, 2004; Cullen and Minor, 2002; Jamali et al., 2009; Brooks and Cullen, 2013), the cerebellum is thought to be a key region of reafference computation in the vestibular system (Angelaki and Cullen, 2008; Cullen, 2011). To better understand IDP during balance mastery, we developed a “dowel balance assay” (Figure 1A), a motor learning task of balance maintenance in freely moving rats. During the assay, a rat was placed on a short dowel 80 cm above the floor while video cameras recorded its behavior. Simultaneously, we recorded PC spike trains from its cerebellar vermis using a wireless system. To avoid falls, the rat must learn to actively counter unbalanced torque by constantly shifting its body in the presence of gravity. With the dowel balance assay, we characterized responses of PCs during those physical activities that involve both active and passive movements, and analyzed IDP shown in their responses.

## Materials and methods

### Animals

Eighteen adult, female Long-Even rats (8–22 weeks, 270–450 g) were used in this study. Rats were housed individually in the animal facility in the Life Science Building at National Taiwan University, Taipei. They were maintained under a 12-h dark/light cycle (lights off at 6:00 p.m.) at 22°C with food and water available *ad libitum*. All experimental procedures were approved by the Institutional Animal Care and Use Committee (IACUC) of National Taiwan University according to guidelines on the use of experimental animals established by the Council of Agriculture, Taiwan.

### Surgery for electrode implantation

Rats were initially sedated with isoflurane in a mixture of oxygen and air, and subsequently anesthetized by *i.p*. injection with a ketamine-xylazine mixture (87 mg/kg ketamine and 13 mg/kg xylazine, mixed before use). Body temperature was maintained at 37.5°C with a heating pad. The head of the rat was immobilized in a stereotactic frame. After the fur was shaved, the epicranium was incised to expose the skull over both hemispheres. Four stainless steel screws were driven into the skull as anchors. One or two craniotomies 1 mm in size were performed at 10.6 and/or 13 mm posterior to the bregma near the midline. The dura matter was opened using a 27-gauge syringe needle to expose lobules V and VI of the cerebellar vermis.

Bundle-type recording electrodes (Tseng et al., 2011) were fabricated with 8 tungsten microwires (0.0014 inch dia.; California Fine Wire Co., Grover City, CA) and a stainless steel microwire (0.002 inch dia.; California Fine Wire Co., Grover City, CA) as reference. Such electrodes were attached to custom-made microdrives, which were stereotactically implanted at 10.6 and/or 13 mm posterior to the bregma in the midline with the tips of the recording electrodes protruding 2.5 mm below the brain surface (located in lobules V or VI). Moving parts of the microdrives were covered with Vaseline and the parts of the microdrive assemblies above the skull were protected by plastic enclosures. The attachment points of these assemblies on the skull were then reinforced with dental cement. After surgery, 5% lidocaine cream was applied to the epicranium. The incision was closed and treated with antibiotic cream.

### Behavior training and test

One and three days before electrode implantation surgery and 7 days after the surgery, behavior training was carried out during the dark period (6:00–7:00 p.m.) with lights on. A rat was brought to the behavior training room in its home cage. After 5 min of adaptation, the rat was taken out of the home cage and placed on a wooden dowel (8 cm long and 2 cm in diameter) suspended 80 cm in the air. The floor underneath was covered with a 5-cm thick layer of bedding material (Figure 1A). After 30 s of on-the-dowel training and regardless of any falls during the 30 s, the rat was returned to its home cage for at least 5 min of rest. After resting, training resumed. Training was considered complete when a rat was able to successfully balance on the dowel without falls for 30 s twice in a row. For training after electrode implantation, if the rat fell, the human trainer caught it to prevent a hard landing that could have displaced the implanted electrodes.

**Figure 2:**
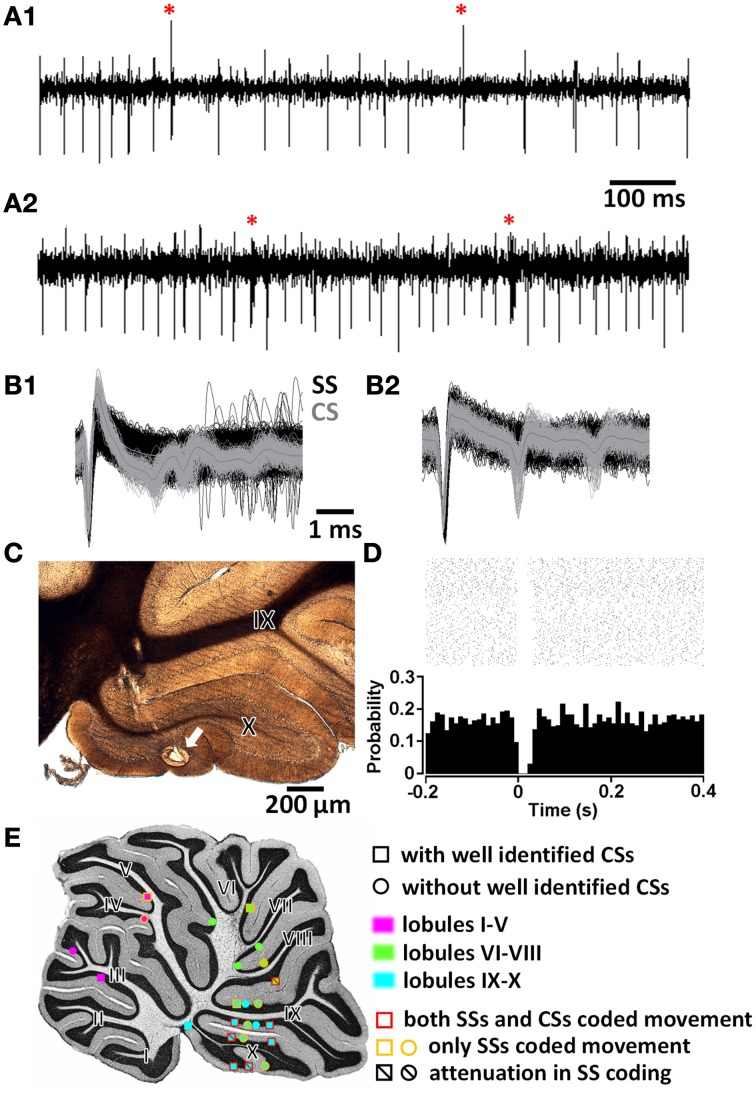
**Purkinje cell (PC) identification. (A1,A2)** Two examples of PC spike trains with characteristic simple spikes (SSs) and complex spikes (CSs, marked by red stars). **(B1,B2)** Waveforms of SS (black) and CS (gray) in expanded time scale. **(C)** Histological confirmation of the recording site (arrow) of the PC in **(A1,B1)**. **(D)** Peri-event raster and histogram showing the brief post-CS pause in SS trains of the PC in **(A1,B1)**. **(E)** Summary of all PC recording sites and their properties in the medial sagittal plane. I—X, lobules I to X of the cerebellar vermis.

Eight days after surgery, behavioral tests were carried out during the dark period (6:00 p.m.–12:00 a.m.) with lights on. After the rat was anesthetized with isoflurane, wireless receivers (Fan et al., 2011) were connected and hex keys were fixed on the hex socket screws on the microdrives. The rat was then placed in a transparent behavior chamber (20 × 26 × 35 cm) beside the balance apparatus, and single-unit activities with characteristic waveforms indicating SSs and CSs of PCs were searched by slowly advancing the recording electrodes down across lobules with the microdrive (maximum ~4 mm ventral to the original site implanted during the surgery). Thirty min after awakening from gas anesthesia, the testing session was video-taped with CinePlex Studio (Plexon) at 80 frames/s. Video-taping was carried out with dual recording from the left side as well as from the anterior aspect, along with neuronal activity. Recorded signals were monitored with SortClient (Plexon) and Chart 5 (ADinstruments). Action potentials from electrophysiological signals were amplified 1000–9000x with high-pass filtering at 0.25 kHz. The resultant waveform was sampled at 40 kHz. Single PC units (signal-to-noise ratio ≥8) were typically identified by the presence of both SSs and CSs (Figures 2A,B). After 10 min of pre-balancing session in the chamber, the rat was placed on the dowel. After the 5-min recording of spontaneous behavior on the dowel, 10 unexpected perturbations (5 brief backward-to-forward shakes of the balance apparatus followed by 5 brief forward-to-backward shakes at intervals of at least 10 s) were introduced. The rat was then returned to the chamber. Cotton balls soaked with isoflurane were placed in the chamber and the top of the chamber was closed. To keep the rat anesthetized after it was sedated, a ketamine-xylazine mixture (87 mg/kg ketamine and 13 mg/kg xylazine, mixed before use) was injected *i.p*. Neuronal activity was recorded for 20 min under anesthesia. To test vestibular responses under anesthesia, rats were fixed on a wood plank during this 20-min recording. Vestibular responses to pitch rotation were tested during the last 5 min of recording under anesthesia.

### Histological analysis

Before sacrificing a rat, recording sites were marked by passing an anodal direct current of 15 μA for 10 s, 3 times. If 2 PCs were recorded in the same rat, the second PC recording site was marked by passing 10 μA for 10 s once. The rat was then deeply anesthetized with >65 mg/kg sodium pentobarbital *i.p*. and perfused transcardially. Perfusates in a three-step procedure included (1) cold 0.9% sterile saline with 0.5% sodium nitrate and 0.5% sodium citrate, (2) cold 4% formaldehyde, and (3) cold 0.1 M phosphate buffered saline (PBS). After perfusion, the brain was removed and stored in 20% sucrose in 0.1 M PBS at 4°C for 2 days. A freezing microtome was used to cut the cerebellum sagittally into 60-μm sections. Lesion sites and electrode tracts were identified and recorded with a microscope (Zeiss Axioplan 2; Cral Zeiss) connected to a digital camera (Nikon) immediately after brain sectioning.

### Data analysis

For behavior analysis, events were extracted from videos using CinePlex Editor (Plexon Inc.). Moving and resting states in the chamber or on the dowel were defined as the periods with and without movement of limbs, head, tail, and trunk, respectively. Breathing, sniffing, whiskering, etc. without gross body motions were considered resting, while moving the head or limbs, grooming, exploring, locomotion, etc. were considered moving. During balancing, because the center of gravity of the rat had to be maintained near a vertical line passing through the center of the dowel if the rat was to stay on the dowel, and because critical vestibular information comes from the vestibular organs, we chose to evaluate the angular aspect of the rat's head in the sagittal plane by recording the angle θ between a vertical line and a line drawn through the center of the dowel and the left eye of the rat (Figure 1B). Angle θ was measured from the video files using PicPick (Daewoong Moon) every 0.2 s when the rat was on the dowel and every 0.1 s during perturbation tests, and only recorded when the nose of a rat was positioned between its two forepaws. Both angular velocity (ω) and angular acceleration (α) in the sagittal plane were calculated using the first and second derivative of θ, respectively. To quantify rat movement on the dowel, absolute values of ω in a period were summed and divided by the length of time (i.e., the time average of ω amplitude, $𝒫\equiv \frac{1}{△\text{t}}\overset{{\text{t}}_{i}+△\text{t}}{\underset{\text{t}={\text{t}}_{i}}{\sum }}|\omega \left(\text{t}\right)|$ for a given initial time point t_*i*_ in a time window with size △t). For each rat, comparable periods with similar motion levels were sorting out with the criteria of those with highest 𝒫 that satisfying (1) at least three of sorted 5–10-s equal-length periods with their differences within 5°/s^2^, (2) at least one of these periods having its midtime within 0–100 s of dowel balancing, and (3) at least one of these periods having its midtime within 100–300 s of dowel balancing. To quantify the magnitude of dowel perturbation, we recorded the swing amplitude of the dowel ϕ (i.e., the angle made by the wires that supported the dowel and a line perpendicular to the ground; **Figure 8A**) and similarly defined it as the sum of the absolute values of the ϕ changing rates (ω_ϕ_) in a period divided by the time length (i.e., $𝓅\equiv \frac{1}{△\text{t}}\overset{{\text{t}}_{i}+△\text{t}}{\underset{\text{t}={\text{t}}_{i}}{\sum }}|\omega_{\phi }\left(t\right)|$). In addition, a loss of footing was defined as the time point at which a rat's hindpaws started to move backward and recorded using tanaMove (http://www.nig.ac.jp/labs/MGRL/tanaMove.htm).

To analyze neuronal activity, spike trains recorded with Chart 5 (ADinstruments) were imported into Offline Sorter (Plexon) to isolate SSs and CSs. Mean firing frequency 〈*f*〉 = 〈ISI〉^−1^, where ISI is the interspike interval, the ISI coefficient of variation $\text{CV}=\frac{\sigma_{\text{ISI}}}{\langle \text{ISI}\rangle }$, where σ_ISI_ is the standard deviation of ISI, and the ISI coefficient of variation of adjacent intervals ${\text{CV}}_{2}=\frac{2}{N-1}\overset{N-1}{\underset{n=1}{\sum }}\frac{\left|{\text{ISI}}_{n+1}-{\text{ISI}}_{n}\right|}{{\text{ISI}}_{n+1}+{\text{ISI}}_{n}}$ were all calculated for different behavioral and physiological states (i.e., resting and moving in the chamber or on the dowel, as well as under anesthesia). Head movement was defined as *I* = θ∨ω∨α. The correlation between SS firing frequency ($f_{SS}=\frac{n_{ss}}{△\text{t}}$, where *n*_*ss*_ is the number of SSs in the time window with length △t) and *I* were analyzed in the *f*_*SS*_-*I* plots. To examine the correlation between CS activity and head movement, we overcame the problem of low and variable firing frequency of CSs by taking the ratio of the CS number (*N*_*CS*_) to the frame number (*N*_*frame*_) in an interval of 𝕀 as a representation of the level of CS discharge during this interval (i.e., $L_{CS}\left(𝕀\right)\equiv \frac{N_{CS}\left(𝕀\right)}{N_{frame}\left(𝕀\right)}$ for 𝕀 = {*I*|*I*∈[*I*_*i*_, *I*_*i*_ + Δ*I*)} with given initial value *I*_*i*_ in an interval of size Δ*I*. Sample data of *N*_*CS*_(𝕀), *N*_*frame*_(𝕀), and *L*_*CS*_(𝕀) with *I* = ω is shown in **Figures 5A–C**, respectively). Corresponding *I* for each CS was given by the interpolated value calculated from behavior records. To quantify the coding capability of SS firing, we first created an SS information association index, defined as the slope between a data point and the data point at the next time point in the *f*_*SS*_-*I* plots (i.e., $ℰ\left(\text{t}\right)\equiv \frac{f_{\text{SS}}\left(\text{t}+1\right)-f_{\text{SS}}\left(\text{t}\right)}{I\left(\text{t}+1\right)-I\left(\text{t}\right)}$). If *f*_*SS*_ consistently had a linear correlation with *I*, then ℰ values would be close. By contrast, if *f*_*SS*_ did not have a linear correlation with *I*, then ℰ values would vary wildly (**Figure 6A**). Therefore, we took the inverse of the standard deviation of ℰs (σ_ℰ_) during a time period (i.e., $𝒲\left(𝕥\right)\equiv \frac{1}{\sigma_{ℰ}\left(\text{t}\right)}$ for t = {*t*|*t*∈[t_*i*_, t_*i*_ + Δ*t*)}) as a quantitive representation of information association capability. To pool data with different units of 𝒲, normalization was accomplished by calculating the z-score of 𝒲 (i.e. *z*_𝒲_(𝕥) $\equiv \frac{𝒲\left(𝕥\right)-\langle 𝒲\rangle }{\sigma_{𝒲}}$, where 〈𝒲〉 and σ_𝒲_ are the mean and standard deviation of total 𝒲s of each PC).

Numerical data were analyzed using custom programs written in Visual C# (Microsoft), PowerBuilder (Sybase, SAP), or MATLAB (MathWorks). Statistical analysis was carried out with Excel (Microsoft) or SigmaStat (Systat Software Inc.). Results were presented as the mean ± standard error of mean. Two-tailed independent Student's *t*-tests or Mann-Whitney rank sum tests were used to test differences between two sets of data that passed or failed the normality test, respectively. To test differences among at least three groups, One-Way ANOVA and Kruskal-Wallis One-Way ANOVA were used.

## Results

### Motor learning measured by head-body pitch angle

We started with a protocol for motor learning with the objective to challenge the rats in a task that was not part of their routine motor repertoire. Thus, the dowel in the balance apparatus was designed to challenge the rat to successfully manage the pitch angle of its head-body in order to stay balance on the dowel (Figure 1A). Rats usually managed to balance by adjusting or moving their heads and bodies, including their tails, mostly in the sagittal plane (Figure 1B). We therefore chose to focus on the head-body pitch angle θ defined as the angle between a vertical line and the line bounded by the center of the dowel and the eye of the rat (Figure 1B, also see Methods). In a biomechanical sense, this pitch angle θ conveyed information on a rat's upper body, which should play a major role in balancing on the dowel. Data on the head-body pitch angle θ was obtained via frame-by-frame video analysis. The angular velocity (ω, Figure 1C) and angular acceleration (α, not shown) was next derived. Fluctuations or variations in ω decreased during the period of motor learning (Figure 1C). During motor learning, the amplitude of rat head-body movement 𝒫 (see methods) decreased significantly (*p* = 0.005, *n* = 18 rats, Kruskal-Wallis One-Way ANOVA, Figure 1D). In addition, we also recorded loss-of-footing events (see Methods). These events became less frequent during the dowel balance task (*p* < 0.001, *n* = 18 rats, Kruskal-Wallis One-Way ANOVA, Figure 1E). These results suggest that rats learned to stay balanced on the dowel with a reduction in head-body movement that can be measured by changes in ω and in 𝒫.

### PC firing in various behavior states

**Figure 3:**
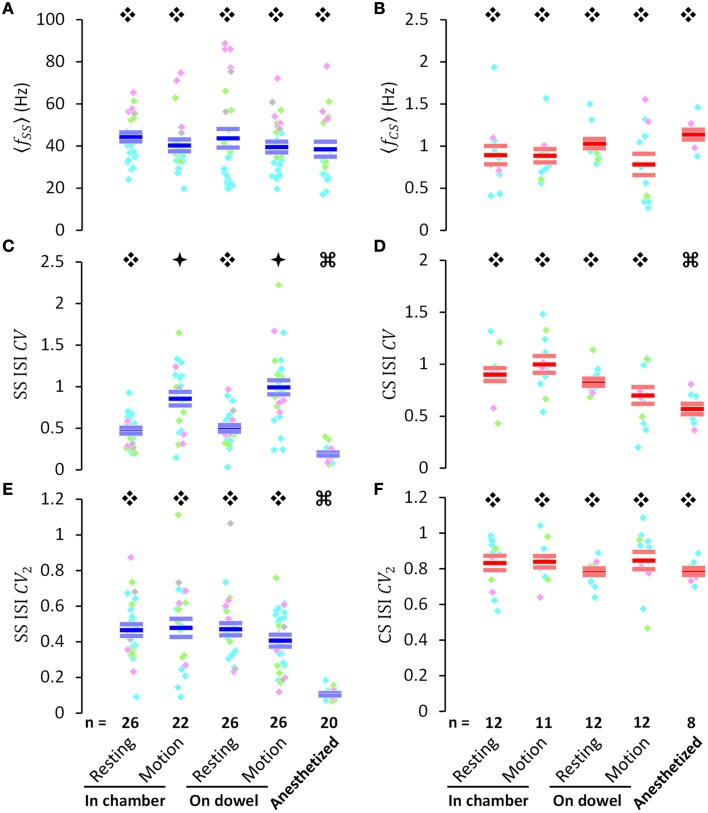
**PC activities in various behavioral and physiological states. (A,B)** Average firing frequency of simple spikes (〈*f*_SS_〉) and complex spikes (〈*f*_CS_〉), respectively. **(C,D)** the coefficient of variation of inter-spike intervals of simple spikes (SS ISI CV) and complex spikes (CS ISI CV), respectively. **(E,F)** the CV of adjacent intervals of simple spikes (SS ISI CV_2_) and complex spikes (CS ISI CV_2_), respectively. All data **(A–E)** were presented for various behavioral or physiological states as indicated in the bottom of the figure. Within each column of colored dots, each individual dot indicates a single PC (pink, PCs in lobules III–V; bright green, PCs in lobules VI–VIII; cyan, PCs in lobules VI–VIII; gray, PCs without histological identification of their recording sites). The three short horizontal bars within each column of color dots denote Mean ± SEM [blue bars are used for SS in **(A,C,E)**, and red bars are used for CS in **(B,D,F)**]. Data marked by different symbols (Ę, ė, or Ģ) in each plot indicate that there is a statistically significant difference between these data (*p* < 0.05, by Student's *t*-test if data passed the normality test, and by Mann-Whitney rank sum test if not). PC numbers are labeled on the bars in **(E,F)**. These results indicate that SS regularity [SS ISI CV in **(C)**] is movement-dependent. Note that the periods of movement and rest were not in equal duration.

We next focused on the discharge pattern of Purkinje cells (PCs), since they are the output cells of the cerebellar cortex. PC discharges consist of two kinds of spike activities: simple spikes (SSs; fast intrinsic spiking modulated by synaptic inputs from cerebellar cortical interneurons) and complex spikes (CSs; a combination of full action potential and additional spikelets on SS trains triggered by strong inputs from a climbing fiber). PCs were identified based on (1) a characteristic waveform of SSs and CSs (Figures 2A,B), (2) a post-CS pause in the SS firing train (Figure 2D), and (3) a recording site being histologically confirmed to be within the Purkinje layer of the cerebellar cortex (Figure 2C). Twenty-six putative PCs were recorded in the cerebellar vermis. During the histological processing of brain tissue, sections containing two PCs were lost. The majority (*n* = 14∕24) were located in the uvulonodular lobe of vestibulocerebellum (lobules IX and X of the cerebellar vermis) (Figure 2E). Among the 26 putative PCs, CSs were unequivocally identified in 12. To be rigorous, our data is based upon 12 PCs and an additional 14 putative PCs.

Because data collected from putative PCs was recorded during different behavioral and physiological states which could have affected PC firing patterns, we next investigated both SS and CS spiking during movement and/or when the rat was resting in the observation chamber, on the dowel, as well as under anesthesia (Figure 3). Mean firing frequencies of both SSs and CSs were not significantly different between moving and resting states in the observation chamber (SS: 44.4 ± 2.2 Hz in resting vs. 40.1 ± 3.2 Hz in motion, *p* = 0.257, Student's *t*-test; CS: 0.89 ± 0.12 Hz in resting vs. 0.89 ± 0.08 Hz in motion, *p* = 0.926, Mann-Whitney rank sum test) or on the dowel (SS: 43.9 ± 4.7 Hz at rest vs. 39.5 ± 2.8 Hz in motion, *p* = 0.430, Student's *t*-test; CS: 1.03 ± 0.06 Hz at rest vs. 0.78 ± 0.13 Hz in motion, *p* = 0.230, Mann-Whitney rank sum test) (Figures 3A,B). Mean firing frequencies also showed no difference in either the resting (SS: *p* = 0.313, CS: *p* = 0.149; Mann-Whitney rank sum test) or moving state (SS: *p* = 0.893, Student's *t*-test; CS: *p* = 0.507, Mann-Whitney rank sum test) between rats in the observation chamber vs. balancing on the dowel. Furthermore, PCs showed no difference in SS or CS firing rates under anesthesia (SS: 38.7 ± 3.3 Hz, *p* = 0.159; CS: 1.13 ± 0.05 Hz, *p* = 0.131; compared with firing rates at rest in the chamber; Student's *t*-test) (Figures 3A,B). Nevertheless, lobule-related differences were found in SS mean firing frequencies (Figure 3A; lobule III–V: 65.1 ± 3.0 Hz, lobule VI–VIII: 45.3 ± 1.8 Hz, lobule IX–X: 30.9 ± 0.9 Hz). These results suggest that mean firing frequencies for CS and SS are not able to differentiate between different behavioral states.

Although mean firing frequencies showed no significant difference for either SSs or CSs under different behavioral and physiological states, the coefficient of variation of SS interspike intervals (ISIs) ($\text{CV}=\frac{\sigma_{\text{ISI}}}{\langle \text{ISI}\rangle }$, calculated as the ratio of standard deviation to mean from the entire spike train, is a time-independent measurement of the variance of a data set) increased significantly during movement, compared with resting states in the observation chamber (0.47 ± 0.04 during resting vs. 0.86 ± 0.08 during movement, *p* < 0.001; Mann-Whitney rank sum test) and on the dowel (0.50 ± 0.05 while resting vs. 0.99 ± 0.10 while in movement, *p* < 0.001; Mann-Whitney rank sum test) (Figure 3C). No difference was found between the CVs of SS ISIs at rest (*p* = 0.740, Student's *t*-test) or in motion (*p* = 0.299, Student's *t*-test), whether the rats were simply in the chamber or balancing on the dowel. CVs of both SS and CS ISIs significantly decreased after anesthesia (SS: 0.47 ± 0.04 at rest in the chamber vs. 0.19 ± 0.02 under anesthesia, *p* < 0.001; CS: 0.90 ± 0.01 at rest in the chamber vs. 0.58 ± 0.04 under anesthesia, *p* < 0.001; Mann-Whitney rank sum test) (Figures 3C,D). Comparatively, this tendency in behavioral states was not evident in the coefficient of variation of adjacent intervals (${\text{CV}}_{2}=\frac{2}{N-1}\overset{N-1}{\underset{n=1}{\sum }}\frac{\left|{\text{ISI}}_{n+1}-{\text{ISI}}_{n}\right|}{{\text{ISI}}_{n+1}+{\text{ISI}}_{n}}$, calculated as the average of ratios computed from two temporal adjacent ISIs, is a measurement of how smooth or abrupt the data set evolved through time) of PC spiking (Figures 3E,F). These results suggest that SS firing pattern changed during movement (i.e., high CV during movement), but this change of the firing pattern was smooth, systematic, and non-random (i.e., consistent level of CV_2_ between resting and motion), indicating that PC SS spikes may dynamically encode ongoing information.

### Information coding in PCs

**Figure 4:**
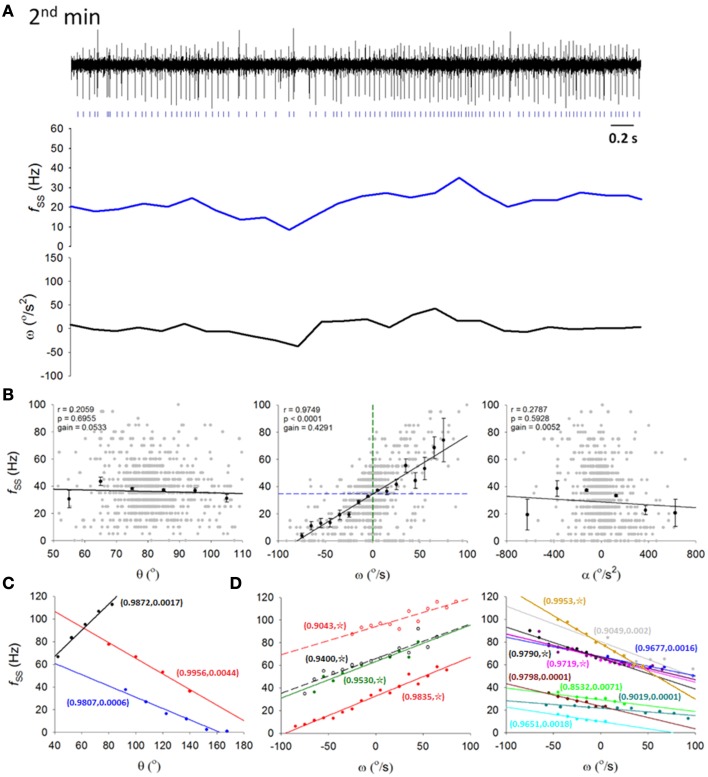
**Information coding in simple spikes. (A)** A 5-s recording from a typical PC SS spike train (top trace) in the second of the 5 min on the dowel with its ongoing firing frequency *f*_*SS*_ (middle trace) and ω (lower trace) calculated from 200-ms time bins. Signs of correlation between *f*_*SS*_ and ω was apparent. **(B)** Correlation between the PC *f*_*SS*_ in **(A)** and the head-body pitch angle (θ, left panel), the angular velocity (ω, middle panel), and the angular acceleration (α, right panel). Gray dots, raw data on θ, ω, and α; black dots and error bars, mean and SEM of θ, ω, and α; black straight lines, results of regression analysis showing the best linear fits of θ, ω, and α; green and blue dashed line in middle panel (*f*_*SS*_-ω plot), ω = 0°/s and the averaged *f*_*SS*_ = 34.61 Hz, respectively. **(C,D)** Data from other 16 PCs with their *f*_*SS*_ encoding either θ **(C)** or ω **(D)**. Paired numbers show (r,p) of each PCs (☆, *p* < 0.0001). These results show that *f*_*SS*_ is linearly correlated with the pitch angle or angular velocity of the head-body movement in the sagittal plane.

The idea that SS may code information in real time is entirely consistent with the observations that there were irregularities or variations in PC spike trains and that such variations with smooth transitions exhibit differences in different behavioral states. It is logical next to inquire exactly what aspect of the rat's movement is encoded by the PC spike train. Since we have shown that the head-body angular velocity ω exhibit significant changes as a consequence of motor learning (Figure 1D), we decided to inquire whether PC spike train may code the head-body angle θ and its derivatives with respect to time, i.e., ω and α. A cursory examination of original traces of a typical single PC revealed potential correlation between the rate of SSs (*f*_*SS*_) and ω (Figure 4A). With a monotonic turn at ω = 0 preferring the downward head motion, *f*_*SS*_ can be shown to linearly encode ω (*r* = 0.9749, gain = 0.4291 Hz/(°/s); *p* < 0.001, One-Way ANOVA; r, square root of the coefficient of determination; gain, the slope). For this particular PC, the correlation between *f*_*SS*_ and ω was strong, but the *f*_*SS*_ correlation with θ or α was weak (Figure 4B).

Among the 26 recorded putative PCs, SS firing rates of 17 cells in various lobules (lobules V–X) of the cerebellar vermis are linearly correlated with θ (Figure 4C) or ω (Figure 4D) of the head (|r| = 0.9832±0.0010; *p* < 0.005, One-Way ANOVA). In most PCs (*n* = 14 of 17), f_SS_ was linearly correlated with ω, while the remainder (*n* = 3 of 17) were linearly correlated with θ. Bidirectional responses were observed in these *f*_*SS*_, with 2 classes of preferred response direction (i.e., head-swing in downward or upward directions). Correlations between *f*_*SS*_ and ω were not significantly different between preferred and non-preferred directions (|r| = 0.9718±0.0033 for preferred direction vs. |r| = 0.9682±0.0001 for non-preferred direction; *p* = 0.629, Mann-Whitney rank sum test). In addition, there also was no significant difference between the gains of these 2 classes of ω-encoding cells (0.350 ± 0.024 Hz/(°/s) for downward-preferred cells vs. 0.364 ± 0.018 Hz/(°/s) for upward-preferred cells; *p* = 0.659, Student's *t*-test). Therefore, our results here suggest that *f*_*SS*_ of some PCs linearly encode head-body movement in the sagittal plane, which was characterized by different degrees of irregularities in different behavioral states. This observation allowed us to relate our electrophysiological data to motor learning data.

**Figure 5:**
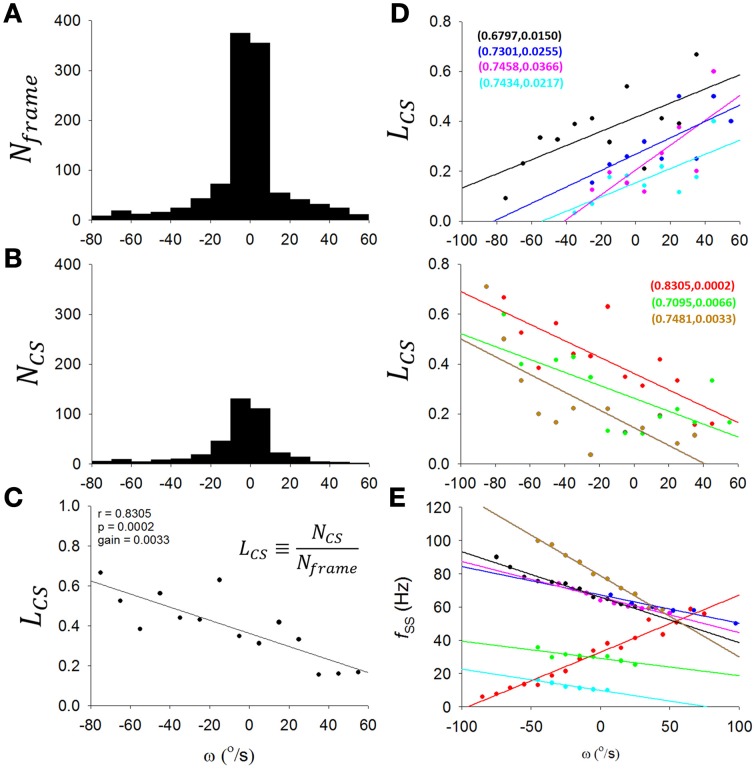
**Information coding in complex spikes. (A)** A histogram of *N*_*frame*_ (number of recorded video frames) as a function of ω (head angular velocity) for a rat. The histogram has a largely symmetrical appearance about zero ω, indicating that the video caught the rat spending most of the time on the dowel with head angular velocity close to zero. **(B)** Histogram of *N*_*CS*_ (number of CSs) as a function of ω for a sample PC of the rat. The histogram also has a largely symmetrical appearance about zero ω, showing that most of the CSs were observed while the head velocity close to zero. **(C)** To determine whether CS activity of this sample PC may indeed be coding ω, we examined the possibility that the apparent symmetrical patterns about zero ω in **(A,B)** may contain some subtle differences to indicate that the PC CS activities in fact show systematic differences with the head motion as a function of ω. This was accomplished by the derivation of **(C)**. Here we calculated for the selected PC a value *L*_*CS*_, a representation of the CS discharge level expressed as the ratio of *N*_*CS*_ [from **(B)**] to *N*_*frame*_ [from **(A)**] in each interval of ω. For this example PC, CS activities show a clear trend for coding ω. **(D)** Such form of CS coding ω was observed in 7 out of 12 PCs in the present study, with either downward-preferred [upper part in **(D)**] or upward-preferred [lower part in **(D)**] pitch movement of the head-body. The sample PC in **(A–C)** is included in the lower part of **(D)** as the red line. **(E)** A *f*_*SS*_-ω plot for the 7 PCs in **(D)**. Each PC was indicated by the same color code as in the two parts of **(D)**. Paired numbers show (r,p) of each PCs. These results suggest that PCs with ω-coding SS may also encoded ω using CS in either a complementary [*n* = 2 of 7, i.e., the lines colored with wood brown and bright green in **(D,E)**] or a reciprocal manner (*n* = 5 of 7, i.e., all other lines).

To overcome the problem of low and variable firing frequency of CSs, we took the ratio (*L*_*CS*_) of the CS number (*N*_*CS*_) to recorded frame number (*N*_*frame*_) in a range of θ, ω, or α as a representation of the level of CS discharge in this interval of θ, ω, or α (Figures 5A–C). To account for the effect of head motion distribution (an example is shown in Figure 5A) on CS distribution (a sample PC recorded from the same rat as Figure 5A is shown in Figure 5B), the number of CSs in each ω interval was divided by the recorded frame numbers of head motion in the same ω interval, i.e., the *L*_*CS*_ (Figure 5C). To be specific, in Figure 5A, we made a histogram plotting the number of recorded video frames (y-axis) with head angular velocity ω (x-axis) for the exemplar rat. The bell-shaped distribution peaked around zero ω suggested that the video caught the rat spending most of its time on the dowel with a head angular velocity close to zero. Similarly, Figure 5B shows the same type of histogram plotting the number of complex spike (y-axis) with head angular velocity ω (x-axis) for the sample PC. A similarly bell-shaped distribution to that in Figure 5A indicates, perhaps not surprisingly, a tendency for complex spikes to occur in periods when head angular velocities were close to zero. If CS activities indeed contains information on head angular velocity ω, then there should be subtle but systematic differences between the two bell-shaped histograms in Figures 5A,B. To represent the conditional probability of observing a CS event in each ω interval, we calculated the ratio of frame number to CS number (y-axis, Figure 5C) with respect to each interval of angular velocity for the selected sample PC (x-axis, Figure 5C). This is not only a normalization process accounting for the fact that most of the data will be from cases with ω close to zero, but also has the added advantage to allow us to overcome a common technical problem in the numerical analysis of low and variable firing frequency of CS. Accordingly, we took the ratio (*L*_*CS*_) of the CS number (*N*_*CS*_) to recorded frame number (*N*_*frame*_) in a range of ω as a representation of the level of CS discharge in this interval of ω (Figure 5C).

In this way, we show that seven of 12 PCs encoded ω with CS discharges. In the upper and lower part of Figure 5D, we show 4 PCs with positive slopes and 3 other PCs with negative slopes; |r| = 0.7938±0.0386, gain = 0.37 ± 0.06%/(°/s); *p* < 0.05, One-Way ANOVA). In addition to Figure 5D, we show in Figure 5E that these PCs with ω-coding CS also encoded ω using SS in either a complementary (*n* = 2 of 7, i.e., lines colored with wood brown and bright green in Figures 5D,E) or a reciprocal manner (*n* = 5 of 7, i.e., all other lines). Therefore, our results here suggest that, not only *f*_*SS*_ of some PCs, CS can also encode head-body movement in the sagittal plane.

### PC information coding attenuated during learning process

**Figure 6:**
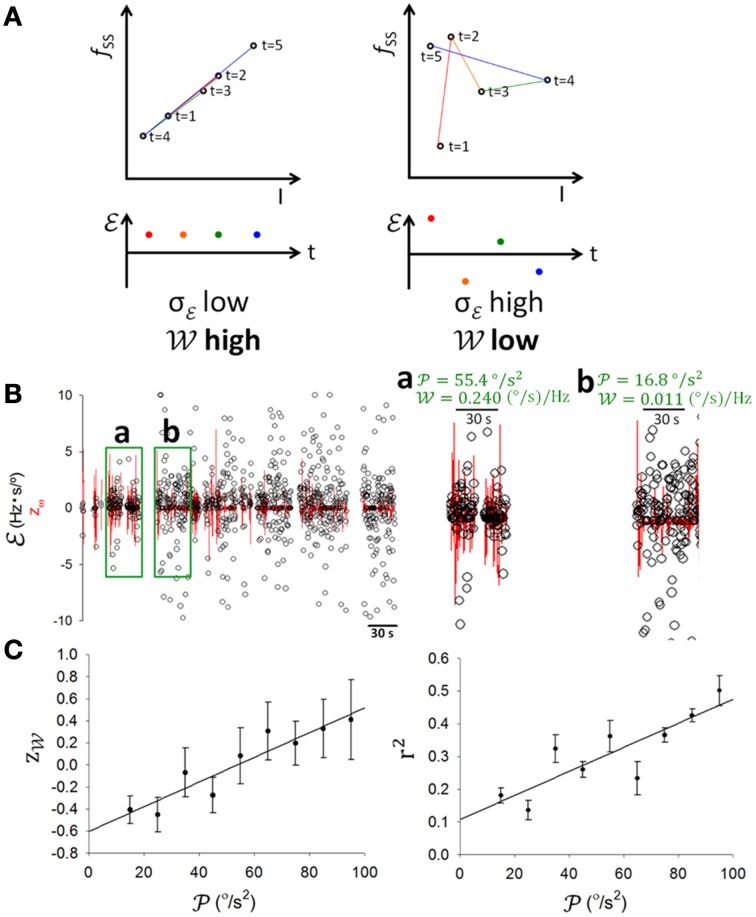
**SS information coding and behavioral activity. (A)** Schematic illustrations of good (upper left panel) and poor (upper right panel) SS information coding association and of high 𝒲, low σ_ℰ_ (lower left panel) and low 𝒲, high σ_ℰ_ (lower right panel) cases, where σ_ℰ_ is the standard deviation of information association index ℰ (t) which is defined as$\frac{f_{\text{SS}}\left(\text{t}+1\right)-f_{\text{SS}}\left(\text{t}\right)}{I\left(\text{t}+1\right)-I\left(\text{t}\right)}$ with *I* depicting θ or ω, and information association capability 𝒲 is derived as $\frac{1}{\sigma_{ℰ}}$. **(B)** Examples with low variations in ℰ accompanied by high 𝒲 and high 𝒫 (a), and high variations in ℰ accompanied by low 𝒲 and low 𝒫 (b). **(C)** Pooled data showing that both *z*_𝒲_ (z-score of 𝒲) and the coefficient of determination r^2^ in *f*_*SS*_-*I* plots correlated positively with 𝒫. This result shows that SS coded information better when there was more movement.

Having established that PCs encoded information on θ and ω (Figures 4, 5), clearly, information of θ and ω would be important to balancing on the dowel. As such information underwent significant changes with motor learning (Figure 1D), our next analysis was to test whether information coding in SS was modulated as a result of motor learning. To quantify SS information coding capability to online information, we devised a dynamical information association index (ℰ), defined as the slope between two temporally adjacent data points in the *f*_*SS*_-ω or *f*_*SS*_-θ plots. If *f*_*SS*_ consistently correlated with updated online information of ω or θ, successive values of ℰ should be consistently close or vary within a small margin. By contrast, if *f*_*SS*_ did not follow the updated information well, values of ℰ should vary more (Figure 6A). Therefore, we took the inverse of the standard deviation of ℰ (σ_ℰ_) in a time window as a measurement of the information association capability (i.e., $𝒲\equiv \frac{1}{\sigma_{ℰ}}$). At first look, the variation of ℰ was low and 𝒲 was high (0.240 (°/s)/Hz) during the 30-s period with high 𝒫 (55.4°/s^2^) (Figure 6Ba), and *vice versa* (𝒲=0.011 (°/s)/Hz), 𝒫 = 16.8 °/s^2^; Figure 6Bb). The population result (*n* = 17) revealed a positive linear correlation between the normalized 𝒲 and 𝒫 (*r* = 0.9334; *p* = 0.002, One-Way ANOVA; Figure 6C, left panel). The coefficient of determination r^2^ in *f*_*SS*_-ω plots also showed a positive correlation with 𝒫 (*r* = 0.8547; *p* = 0.0033, One-Way ANOVA; Figure 6C, right panel). These results suggest that both static (i.e., r^2^) and dynamic associations (i.e., 𝒲) of SS to sensory information depend upon motion level.

**Figure 7:**
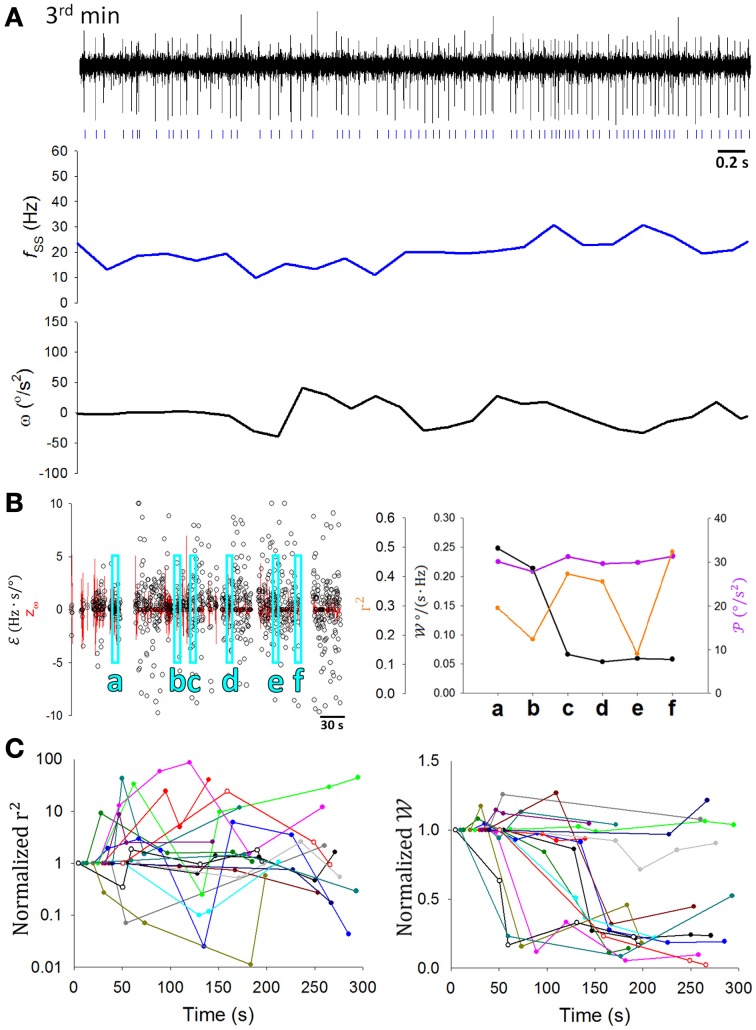
**Attenuation of information association capability during motor learning. (A)** A 5-s recording of the same PC SS activity with its *f*_*SS*_ and ω during the third minute of balancing on the dowel (the corresponding data during the second minute being shown in Figure 4A). Signs of correlation between *f*_*SS*_ and ω was not as apparent as in Figure 4A. **(B)** During periods with comparable movement levels (𝒫 = 30± 2.5°/s^2^) (a–f), 𝒲 decreased suddenly (e.g., in period c). **(C)** The ratio of 𝒲, but not r^2^, to the value in the first period decreased significantly during motor learning for many PCs (*n* = 10 of 17) (the same colored data in left and right part of **(C)** are from the same cell, but the color code bears no relationship to earlier plots). These results show the dynamic nature of the changes in the information association capability 𝒲 during motor learning.

A critical test in the present study was to determine how SS coding of sensory information was modulated as the rats learned to balance on the dowel. Figure 7A shows a typical PC in which the correlation between SS firing and head movement in the third minute on the dowel appeared to be weaker than that in the second minute (Figure 4A, same PC). Changes such as those shown in Figures 4A, 7A were routinely observed in the PCs of the present study. ℰ also seemed to become more variable with training while 𝒲 decreased (Figure 6B). However, since 𝒲 and r^2^ correlated positively with 𝒫 (Figure 6) and 𝒫 decreased during the time on the dowel (Figure 1), the decrease of 𝒲 during balancing may simply be due to the decrease in 𝒫. To reduce the influence of 𝒫 on the association between SS firing and head-body movement, periods with comparable 𝒫 were selected to test the attenuation of their association (Methods; Figures 7Ba–f). r^2^ fluctuated through the periods with comparable 𝒫, however, a sudden drop of 𝒲 occurred during the period on the dowel (Figure 7B, right panel). For the 17 PCs with SSs encoding θ or ω, 10 ω-coding cells became unresponsive with over 50% attenuation in 𝒲 (23.42 ± 0.02%; intercepted 𝒫s were ranged from 20 to 70°/s^2^; Figure 7C).

**Figure 8:**
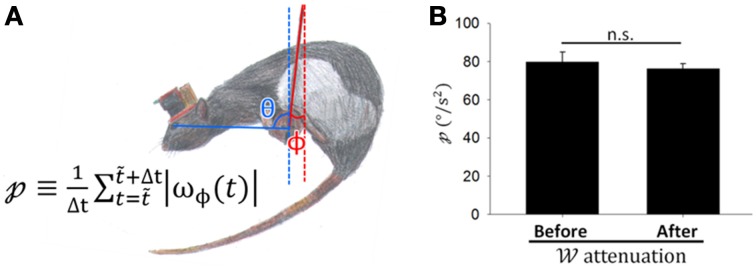
**Coding capability attenuation was not related to less dowel movement. (A)** To quantify the magnitude of dowel perturbation [𝓅; defined by the equation shown in **(A)**], we recoded the swing angle of dowel perturbation ϕ and defined 𝓅 as the sum of the absolute values of the ϕ changing rate (ω_ϕ_) in a period divided by the length of time. **(B)** 𝓅 did not differ significantly before and after attenuation in 𝒲 occurred (periods with comparable movement levels above and below 50% attenuation; *p* = 0.566, Student's *t*-test). This result suggests that coding capability attenuation was not related to a reduction in dowel movement.

In the rostral fastigial neurons of monkeys, active movement was accompanied by weaker sensory responses than passive movements (Brooks and Cullen, 2013). Despite the prevailing view that CS discharge responds to passive motion caused by error events (Ito, 1972), some investigators thought that SS discharges encode errors (Popa et al., 2012, 2013). Perhaps attenuation in 𝒲 in these PCs is caused by decreasing passive movement during the motor learning as a response to state transformation in the weight between active and passive movement. To distinguish directly between active and passive movement in freely behaving animals is challenging; however, we tried to indirectly distinguish components of active and passive motion from video recordings during balancing. When rats moved voluntarily on the dowel, the passive motion component may be represented in perturbations of the hanging dowel. To quantify perturbation of the dowel (𝓅), which might be positively correlated with the level of passive motion, we recoded the swing amplitude of dowel perturbation (Figure 8A) and defined 𝓅 as the sum of the absolute values of the ϕ changing rate during a period divided by the length of the period. 𝓅 was not significantly different before and after attenuations occurred (*p* = 0.566, Student's *t*-test; Figure 8B), indicating that passive motion due to dowel swing was of similar magnitude during these periods. Thus, we demonstrate that attenuation in the 𝒲 of PCs is a result of the plasticity in PC responses to sensory information.

### PC information coding recovered by unexpected perturbation

**Figure 9:**
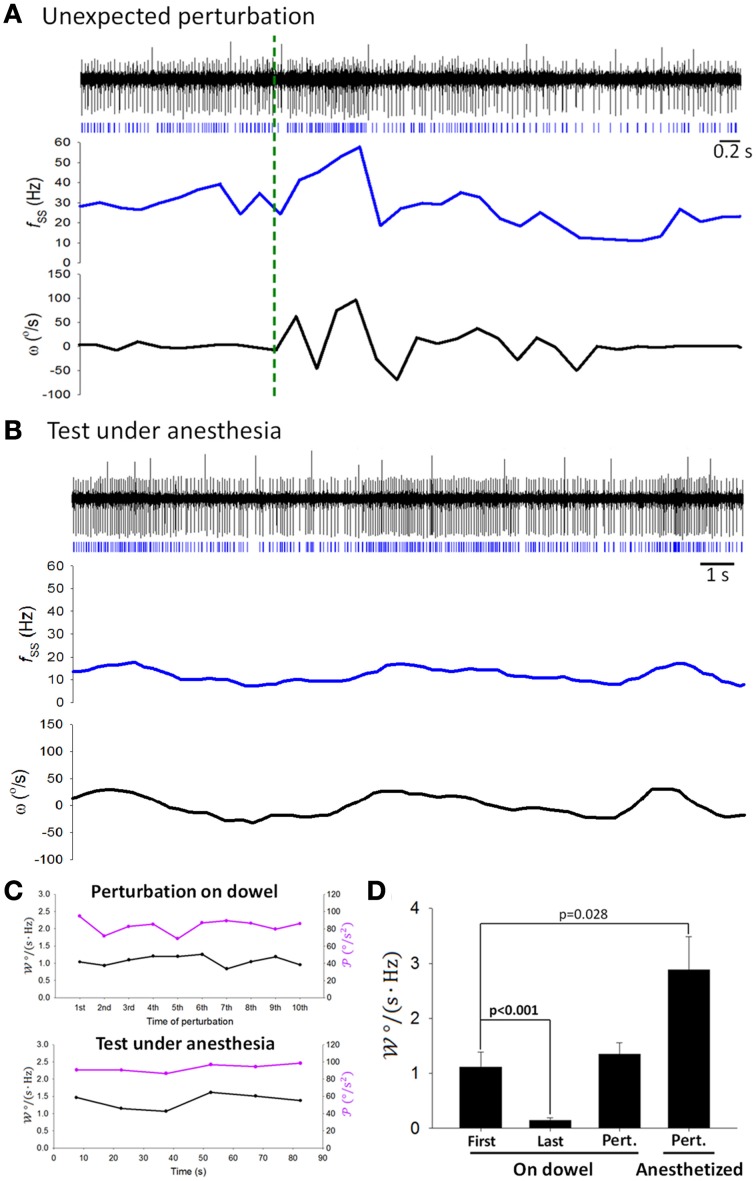
**Coding capability remained the same during passive stimulation**. Simple traces of the same PC (as in Figures 4A, 7A) showed coding of ω during **(A)** additional perturbations (green dashed line shows the starting point of the perturbation) and **(B)** stimulation under anesthesia. **(C)**𝒲 showed no time-dependent change due to passive perturbation (upper panel) or by a sustained vestibular stimulus under anesthesia (low panel). **(D)** 𝒲 tested under different behavioral and physiological states. First, the first analyzed period on the dowel; Last, the last analyzed period on the dowel; Pert.—On dowel, unexpected perturbation when a rat was on the dowel; Pert.—Anesthetized, perturbation when a rat was under anesthesia.

To inquire whether attenuation in SS information coding observed in Figure 7 was indeed related to motor learning, we introduced additional perturbations at irregular time intervals by transiently shaking the balance apparatus after the 5-min recording of the balancing behavior. SS firing responded to head movement during these additional perturbations (Figure 9A), with value of 𝒲 similar to that before response attenuation (1.11 ± 0.27 Hz/(°/s) before attenuation vs. 1.344 ± 0.21 Hz/(°/s) after the additional perturbations, *p* = 0.407, Student's *t*-test; Figure 9D). In addition, sensory responses of SS still encoded head motion under anesthesia (Figure 9B), with 𝒲 maintained at a level higher than that before response attenuation (2.88 ± 0.60 Hz/(°/s); *p* = 0.028, Student's *t*-test; Figures 9C,D). These results suggest that SS information coding attenuation during motor learning may be a selective filtering of reafference information.

### Static correlations between SS and CS firing

**Figure 10:**
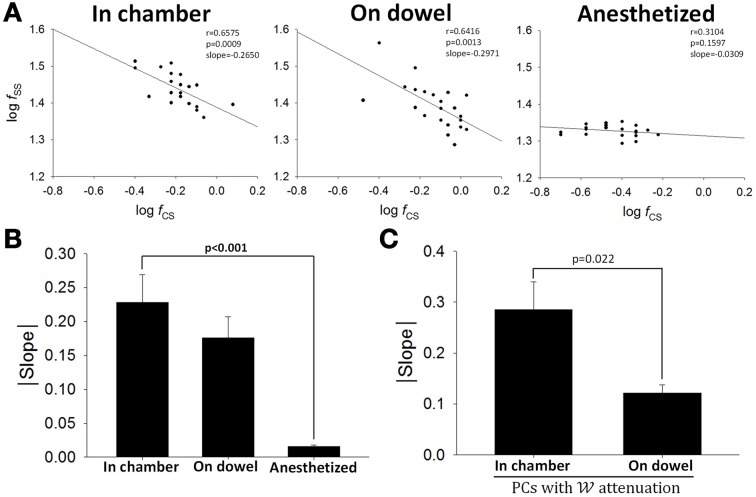
**Interaction between SSs and CSs. (A)** Double-logarithmic plots of SS and CS firing frequency of a PC when the rat was in the chamber (left), on the dowel (middle), and under anesthesia (right). Each data point in **(A)** was the mean firing frequency within a 15-s recording period. **(B)** Absolute values of slopes in the double-logarithmic plots of SS and CS firing frequency under various behavioral and physiological states (in chamber, *n* = 12; on dowel, *n* = 12; anesthetized, *n* = 8). **(C)** SS-CS interaction was lower during balancing for the PCs with 𝒲 attenuation (*n* = 4). These results suggest that the negative correlation between firing frequencies of SSs and CSs observed when rats were awake may contribute to PC plasticity.

We had established previously that the relationship between responsive CS and SS is either a reciprocal or a complementary one (Figures 5D,E). Now using a 15-s time window for averaging, the relationship between firing rates of SS and CS was illustrated in double-logarithmic plots. Figure 10 shows that for a typical PC, significant correlation between CS and SS firing was observed when the rats was in the observation chamber (Figure 10A, slope = 0.228 ± 0.041) or on the dowel (Figure 10B, slope = 0.176 ± 0.031), but not under anesthesia (Figure 10C, slope = 0.016 ± 0.002; *p* < 0.001 compared with data from the chamber, Mann-Whitney rank sum test). However, unlike the two types of relations between SS and CS responses described in Figures 5D,E, only negative correlation was observed in this static analysis. That was the case for the PC shown in Figure 10A and all other PCs (Figure 10B). The observations in Figures 10A,B suggest that the negative correlation between SSs and CSs in seconds time scale could only be observed in awake and behaving rats. To further investigate the role of CS-SS interaction in motor learning, we analyzed the slope of the CS-SS relationship in rats when they were in the chamber vs. those on the dowel in PCs that showed significant attenuated information coding during or as a result of motor learning. Figure 10C shows that the correlation between SSs and CSs was weaker on the dowel compared with that when rats were placed in the observation chamber (slope: 0.285 ± 0.055 in the chamber vs. 0.122 ± 0.016 on the dowel, *n* = 5, *p* = 0.022, Student's *t*-test; Figure 10C). These data indicate that SS-CS interaction may mediate the neural plasticity of PCs in filtering out sensory reafference from movement during motor learning.

## Discussion

In this study, we investigated neural plasticity of cerebellar information processing in rats by examining PC spike trains as the rats performed a motor learning task. Our data show that a sudden attenuation of information association capability occurred in many PCs as the rats learned to stay balanced on the dowel. Specifically, PC responses to active movement were selectively filtered out during this learning process, suggesting the IDP of cerebellar system for reafference learning.

### Information coding of SSs in volitional behaviors

SSs fire spontaneously in a regular manner (Nam and Hockberger, 1997). They have also been shown to encode information regarding intensity, position, direction, and velocity (Harvey et al., 1977; Mano and Yamamoto, 1980; Marple-Horvat and Stein, 1987; Fortier et al., 1989; Fu et al., 1997; Coltz et al., 1999; Roitman et al., 2005; Pasalar et al., 2006; Barmack and Yakhnitsa, 2011; Hewitt et al., 2011; Popa et al., 2012; Brooks and Cullen, 2013). In the present study, we showed irregular firing with smooth changes during motion (Figure 3) with a linear correlation of SS firing frequency and head-body angular velocity (Figure 4). Lobule-related differences were observed in SS mean firing rate (Figure 3A), which may be related to differences in zebrin II expression in the anterior lobe and uvulonodular lobe near the midline of cerebellar vermis (Zhou et al., 2014; Cerminara et al., 2015). Head-body movements encoded by these PCs in our study may result from vestibular [i.e., those in lobules IX–X of the classical vestibulocerebellum (Barlow, 2005)] as well as non-vestibular [i.e., those in lobules V–VIII of the classical spinocerebellum (Barlow, 2005)] information, such as neck muscle tension. In addition, very different lobules of the rat cerebellum also appeared to receive similar projections (Ruigrok, 2003; Voogd et al., 2003; Fujita and Sugihara, 2013; Lee et al., 2014). Regardless of the type of movement-related information encoded by these PCs, SS firing rates of PCs in the present study showed a linear coding response to the head-body angle measure. The same type of correlation has been noted from inputs [e.g., mossy fiber terminals (Arenz et al., 2008)] to outputs of cerebellar cortex [e.g., PCs (Fushiki and Barmack, 1997; Bosman et al., 2010; Badura et al., 2013)], suggesting that this linear relation may be a functional part of information integration of the cerebellar cortex circuitry. More specifically, the linear correlation reported in previous studies was found to be more obvious under slow, strong, periodic stimulation (Barmack and Shojaku, 1995; Yakusheva et al., 2010). Here we further show that the positive correlation between information coding ability and information strength also occurs in non-periodic volitional behavior (Figure 6).

### SS-CS interaction in volitional motor learning

Many investigators thought that the firing frequency of CSs is too low and too variable to encode sensory information (Rushmer et al., 1976; Ebner and Bloedel, 1981; Ito et al., 1982; Llinas and Yarom, 1986; Andersson and Armstrong, 1987). Other investigators, however, have observed information encoding of CS activity over a range of 0.001–1.5 Hz under vestibular or tactile stimulation (Maekawa and Simpson, 1973; Graf et al., 1988; Leonard et al., 1988; Barmack and Shojaku, 1995; Fushiki and Barmack, 1997; Frens et al., 2001; Barmack and Yakhnitsa, 2003; Yakhnitsa and Barmack, 2006; Bosman et al., 2010). In the present study, most (5 of 7) PCs with CS coding head movement encoded ω with SSs in a reciprocal manner, but in 2 PCs, SSs and CSs preferred the same direction (Figures 5D,E). By analyzing 15-s time windows (Figure 10), we further showed that the correlation between firing frequencies of SSs and CSs was observed only when rats were awake. However, the negative correlation between SS and CS rates in 15-s time windows is different from the two type of observed relations between SS and CS responses. These results suggest that the relation between SSs and CSs can vary among different PCs and under different functional perspectives.

Under a vestibuloocular reflex (VOR) learning protocol without climbing fiber instructive signals, the change in the gain of VOR was reduced but not eliminated (Ke et al., 2009). Furthermore, optogenetic activation of SS activities in PCs contributed to the expression of motor learning (Nguyen-Vu et al., 2013). These observations suggest that sensory information conveyed in SS activities may be necessary for motor learning. Recently, climbing fiber activity has been shown to modulate the gain of SS firing in neighboring PCs through the actions of molecular layer interneurons by processes involving synaptic and extrasynaptic spillover mechanisms (Mathews et al., 2012; Coddington et al., 2013). SS-CS interaction was also found to be responsible for PC plasticity during motor learning (Medina and Lisberger, 2008; Yang and Lisberger, 2014). In the present study, we have shown that for PCs with SS coding attenuation, the correlation between SSs and CSs was weaker during balancing (Figure 10C), suggesting that the strength of correlation between SS and CS rates may alter coding ability of learning-required SS responses.

### IDP in reafference learning

PCs have multiple roles in sensorimotor calibration, including prediction, teaching, and command (Medina, 2011; Veloz et al., 2014). Unlike the previous investigation of PC responses during active and passive movement (Bauswein et al., 1983), our data concern the process of learning. In the present study, we found a sudden loss of information association capability 𝒲, but not the coefficient of determination r^2^, in some PCs during balance learning (Figure 7). Single PC is thought to process information from multiple peripheral receptive fields as well as the cerebral cortex (Freeman, 1970; Jörntell and Ekerot, 2002, 2003). At least two issues may be relevant to our observations. First, a given PC may process more information than just the head-body angle measured in the present study (Johnson and Ebner, 2000; Ebner et al., 2011). If so, this could contribute to data scatter (Figure 4B) and could also be reflected in the fluctuation of r^2^ (Figure 7C), while higher PC coding capacity during the test under anesthesia (Figure 9) suggests that PC may convey specific exafferent information (i.e., the head-body angle measured in the present study) rather than movement-related activity. A technical issue here is that the time window used in data analysis relative to the time constant of the underlying neurophysiological processes may become critical. This may be a reason why r^2^ fluctuated through short periods of comparable 𝒫, even the correlation between SS firing and head motion showed in an overall 5-min time windows. Second, assuming the PC output to be a linear summation of multiple responses (Shinmei et al., 2002; Fukushima et al., 2011), if one were to demonstrate how well SSs respond to the changes of certain input, a dynamic measure between temporally adjacent data points, i.e., 𝒲, should be more specific than a mean measure of the correlation between absolute SS firing rate and certain input, i.e., r^2^.

The coding attenuation of PC SS was observed to accompany the learning process, while the precise role of PC in motor learning may be implicit and awaits further investigation. One possibility is that PC SS were parts of the motor coding. As training brought on a state of automaticity in body balance on the dowel, more efficient motor activities actually requiring less PC SS coding. On the other hand, a related and somewhat intertwined possibility is that PC SS may be part of the reafferent responses primarily conveying sensory information. As movements become more routine, attaining a level of automaticity, there is a reduction in such reafferent responses. Our results in PC coding of ω and θ (e.g., Figures 4, 5) as well as the higher level of PC SS coding in the anesthetized state lend more direct support to this possibility. Furthermore, patients with intention tremor, or cerebellar tremor, overshoot or undershoot their intended position of hand, arm, or leg. This dysmetria has also been suggested to be caused by the cerebellar dysfunction in reafference computation (Manto, 2009).

Compared with vestibular sensory responses due to passive motion, vestibular sensory response gains of deep cerebellar nuclear neurons caused by active motion showed a 25–100% reduction (Brooks and Cullen, 2013). In general, Purkinje cells may regulate nuclear cells through three different modes: (1) inverter (Armstrong et al., 1975; Cody et al., 1981; Rowland and Jaeger, 2005), (2) T-type rebound (Llinás and Mühlethaler, 1988; Aizenman et al., 1998; Czubayko et al., 2001; Molineux et al., 2006), and (3) synchrony code (Person and Raman, 2011, 2012). The proportion of PCs responding vs. not responding to active motion may therefore modulate the gain of active motion responses in deep cerebellar nuclear neurons. In addition, the time courses of sudden losses in information association capability varied among PCs (Figure 7C), suggesting that the gradual change in behavior (Figures 1D,E) may be due to the change in population ratio of PCs that responding to active motion. As a result, the filter strength of the cerebellar cortex may be an essential mechanism in reafference learning.

The cerebellar vermis receives dense input from motor cortex, in addition to somatic sensory input from ascending spinal pathways (Coffman et al., 2011; Apps and Watson, 2013). Moreover, these vastly different projectional systems may be organized into sagittal or longitudinal stripes in the cerebellar cortex. For example, Shinoda and Sugihara (2013) reported that spinocerebellar and vestibulocerebellar mossy fibers have a higher tendency to target aldolase C-negative sagittal zones of the cerebellar cortex, while pontocerebellar mossy fibers have a higher tendency to terminate in aldolase C-positive sagittal zones of the cerebellar cortex. It is likely that such an organization may allow information processing on reafference. With parallel fibers that run perpendicularly to the longitudinal plane, this difference in topography of the mossy fiber system suggests two maps of receptive fields integrated in the cerebellar cortex: one of efferent copies from the will center for volitional motions (e.g., the cerebral cortex), and the other of sensory inputs from the external world. Studies in cats showed that PCs could be activated or inhibited by tactile stimuli in different body locations, and that the sizes of these receptive fields were also alterable as a climbing fiber-dependent plasticity in parallel fiber receptive fields (Jörntell and Ekerot, 2002, 2003). With the functional analogy of the internal modeling system (Lisberger, 2009), information monitor (Bower, 2013), or adaptive filter (Dean et al., 2013), cancelation of a specific receptive field response, with the fast learning phase similar to the sudden attenuation in our results, can be accomplished by adaptive filtering in the cerebellar cortex model (Porrill and Dean, 2008). In the dowel balance assay, while the rats were allowed to freely behave on the dowel, they need to balance their bodies to avoid falling down from the dowel. Therefore, neuronal plasticity of reafference computation in this motor learning, i.e., IDP, may result from receptive field plasticity involving integration of the two receptive maps in the cerebellar cortex.

### Conflict of interest statement

The authors declare that the research was conducted in the absence of any commercial or financial relationships that could be construed as a potential conflict of interest.
